# Comparative Efficacy of Fish Meal Replacement With Enzymatically Treated Soybean Meal on Growth Performance, Immunity, Oxidative Capacity and Fecal Microbiota in Weaned Pigs

**DOI:** 10.3389/fvets.2022.889432

**Published:** 2022-05-31

**Authors:** Ning Yang, Mohan Li, Yuetong Huang, Xiaona Liang, Zhizhong Quan, Haiying Liu, Jiantao Li, Xiqing Yue

**Affiliations:** ^1^Animal Food Processing Laboratory, College of Food Science, Shenyang Agricultural University, Shenyang, China; ^2^Liaoning Complete Biotechnology Co., Ltd., Tieling, China; ^3^Animal Nutrition Laboratory, College of Animal Science and Medicine, Shenyang Agricultural University, Shenyang, China

**Keywords:** enzymatically treated soybean meal, fish meal, fecal microbiota, immunity, antioxidant capacity, piglets

## Abstract

This study investigated the growth performance, immunity, antioxidant capacity and fecal microbiota of weaned pigs by partially or completely replacing dietary fish meal with enzymatically treated soybean meal. A total of 144 piglets (initial body weight of 7.19 ± 0.11 kg) weaned at 28 d were allotted to 3 dietary treatments (6 replicates per treatment): 4% fish meal diet (FM); 2% fishmeal plus 6% enzymatically treated soybean meal (ESBM1); and 6% enzymatically treated soybean meal without fish meal (ESBM2). The experimental period was 28 d, serum was collected at day 14 and day 28 for biochemical parameters analysis, feces was obtained for microbiota analysis at 28d. The body weight, average daily gain and average daily feed intake of piglets in the ESBM2 group were significantly increased compared with those in the FM and ESBM1 groups from 0 to 28 d, respectively (*P* < 0.05). The diets with enzymatically treated soybean meal in ESBM1 and ESBM2 groups decreased the diarrhea rate (*P* < 0.05). Compared with FM, ESBM1 and ESBM2 decreased 5-hydroxytryptamine (5-HT) (*P* < 0.05). ESBM1 decreased diamine oxidase (DAO) and Interleukin 6 (IL-6) compared with FM and ESBM2 (*P* < 0.05). ESBM1 decreased serum Interleukin 1β (IL-1β) compared with FM at d 14 (*P* < 0.05). The serum Immunoglobulin E (IgE), secretory curl associated protein 5 (sFRP-5) were higher in ESBM1 compared with FM and ESBM2 (*P* < 0.05). ESBM2 increased super oxidase dismutase (SOD) level and decreased malondialdehyde (MDA) content compared with FM and ESBM1, the concentration of SOD in ESBM1was higher than that in FM (*P* < 0.05). ESBM1 decreased cortisol and caspase 3 (Casp-3) (*P* < 0.05). FM showed a higher content of tri-iodothyronine (T3) (*P* < 0.05) and a lower thyroxine/ tri-iodothyronine ratio compared with those in the other two groups (*P* < 0.05). The concentration of leptin was lower in ESBM2 (*P* < 0.05). ESBM1 had a higher α-diversity than ESBM2 (*P* < 0.05). The microbiota composition was different among three treatments (difference between FM and ESBM1, *p* = 0.005; FM and ESBM2, *p* = 0.009; ESBM1 and ESBM2, *p* = 0.004). ESBM2 tend to increase the abundance of Firmicutes (*P* = 0.070) and decrease Bacteroidetes (*P* = 0.069). ESBM2 decreased the abundance of *Parabacteroides* and increased *SMB*53 compared with FM (*P* < 0.05). The spearman correlation analysis revealed that the abundance of *Parabacteroides* enriched in FM group was negatively correlated with SOD, *Megasphaera* enriched in ESBM2 group were positively correlated with SOD. The abundance of *Lachnospira* enriched in ESBM2 group were negatively correlated with serum concentration of D-lactate, DAO, IL-6, and NO. In conclusion, under the conditions of this study, diet with only ESBM demonstrate the beneficial impact on intestinal microbiota developments, antioxidant capacity as well as growth performance for weaned pigs.

## Introduction

Weaning is the most stressful period for young pigs. Along with changes in the growing environment and feed source, piglets commonly suffer from severe decreases in feed intake and increases in diarrhea (1). The altered gut environment is favorable for some opportunistic pathogens to colonize, resulting in diarrhea (2). Dietary proteins from different sources have different digestibility. Undigestible dietary protein could challenge gut health by abnormal fermentation in the hindgut. Fish meal (FM) is considered a high-quality and highly digestible dietary protein in the immediate postweaning period (3) but is not economical due to its high price and the variation in quality among batches (4).

Soybean meal (SBM) is the most commonly used plant protein due to its relatively balanced amino acids and ease of procurement, but many antinutritional factors (ANFs) limit the utilization of unprocessed SBM by young animals. For instance, glycinin and β-conglycinin can aggravate weaning stress by causing digestive disorders, immune responses, gut morphology impairment, and ultimately restrained growth performance (5, 6). Bioprocessing, such as enzymatic treatment, has been proven to be an effective way of eliminating ANFs, while peptides and amino acids from partially or completely degraded proteins are more easily digested and absorbed (7, 8). The higher digestibility of enzymatically treated soybean meal (ESBM) could decrease the transfer of undigested protein into the hindgut and reduce the production of potentially toxic products from metabolism (9). Accumulated evidence has demonstrated that ESBM exhibits beneficial effects on growth performance and improves intestinal morphology and immune status (6, 9, 10).

The intestinal microbiota is crucial for the maintenance of gut homeostasis, mainly through competitive exclusion of enteric detrimental microorganisms and pathogens (11). Feed digestibility and nutrient absorption are also strongly affected by gut microbes attributed to microbial enzyme production and metabolites. Many commensal microbes existing in the gut, diet and physiological status of animals could change the composition and abundance of commensal microbiota. Nitrogen from different structural dietary proteins is digested in different ways in the intestine, affecting the nutritional supplementation for enteric microflora (12). It is speculated that feed digestibility and intestinal homeostasis are modulated concomitantly when dietary protein sources are changed. Previous research which concerned more effect of different plant-based sources on intestinal microbiota showed that piglets fed 15.1 and 7.8% ESBM during two experimental phases had higher fecal alpha diversity compared with pigs fed equal amount of hydrolyzed wheat protein and fermented soybean meal, enhanced the relative abundance of Bacteroidetes, *Oscillospiraceae*, and *Christensenellaceae*, and decreased the abundance of *Clostridiaceae* in the feces of weaned pigs (9). Another study demonstrated that all of the differently treated SBMs which replacing half of dietary fishmeal increased the fecal *Lactobacillus* counts, while the *Escherichia coli* counts were not affected compared with the fish meal treatment (13).

However, to the best of our knowledge, the unchanged or improved growth performance that results by replacing fish meal with ESBM has not been intensively studied in terms of the gut microbiota and the correlations between serum parameters and intestinal microbiota. In recent years, the 16S rRNA gene amplicon sequencing technique has been widely utilized to investigate microbial composition and diversity (14, 15). Therefore, the objective of this study was to investigate the effects of replacing FM with ESBM on the growth performance, serum parameters indicating oxidative and immune status, metabolism and stress related hormones, microflora composition and diversity in the feces of weaned pigs using Quantitative Insights Into Microbial Ecology (QIIME2) and high-throughput sequencing.

## Materials and Methods

### Animals, Diets and Design

The animal study protocol was approved by the Institutional Ethics Committee of Shenyang Agricultural University Institutional Animal Care and Use Committee (protocol code 2021042567 and April 25th, 2021).

A total of 144 piglets [Landrace ×(Large White × Duroc)] weaned at 28 d with initial body weight of 7.19 ± 0.11 kg were randomly allotted to one of three dietary treatments. Each treatment consisted of 48 piglets, with 6 replicate pens per treatment and 8 piglets (4 male and 4 female) per pen. Piglets in all treatments were housed in pens in an environmentally controlled room with a slatted plastic floor equipped with a one-sided stainless-steel feeder and a nipple drinker for 28 d trial period. Piglets had *ad libitum* access to feed and water throughout the experimental periods. All diets were formulated according to China National Standards of formula feeds for piglets (GB/T 5915-2020) (16). Treatments were as follows: (1) the FM diet contained 4% fish meal (Technológica de Alimentos, S.A., Peru); (2) the ESBM1 diet contained 2% fish meal and 6% enzymatically treated soybean meal (ESBM, Liaoning Complete Biotechnology Co., Ltd., Liaoning, China); and (3) the ESBM2 diet contained 6% enzymatically treated soybean meal (ESBM) without fish meal in the diet. The compositions of Soybean meal, ESBM and fish meal are shown in Table 1. The diet compositions and nutrient levels are shown in Table 2. On day 0, 14, and 28, each piglet was weighed to calculate the average daily gain (ADG), and feed consumption was measured per pen at the same time to calculate the average daily feed intake (ADFI). The diarrhea rate was assessed by an observer and calculated according to Pan's description (17): diarrhea rate (%) = (number of pigs with diarrhea days) / (number of pigs × total observational days) ×100. The scoring system was defined as follows: 1 = hard feces; 2 = slightly soft feces; 3 = soft, partially formed feces; 4 = loose, semiliquid feces; and 5 = watery, mucous-like feces. The occurrence of diarrhea was defined as maintaining fecal scores of 4 or 5 for 2 consecutive days.

**Table 1:** Nutrient value and antinutritional factors of SBM, ESBM and FM (as-fed basis).

**Item**	**SBM**	**ESBM**	**FM**
Dry matter, %	86.98	89.82	91.80
Crude protein, %	45.14	48.26	65.32
Crude ash, %	6.21	6.14	15.60
TCA-SP, %	1.87	18.98	2.73
Amino acids			
Threonine, %	1.72	1.88	2.87
Valine, %	1.70	2.06	3.52
Methionine, %	0.47	0.44	2.01
Isoleucine, %	1.75	2.16	2.76
Leucine, %	3.86	4.32	4.76
Phenylalanine, %	2.33	2.59	2.03
Lysine, %	2.75	2.73	5.37
Serine, %	2.42	2.48	2.83
Glutamicacid, %	8.77	9.46	9.12
Glycine, %	1.82	1.90	4.20
Alanine, %	1.48	1.58	4.62
Cystine, %	1.42	1.46	0.76
Asparticacid, %	5.41	5.81	6.41
Tyrosine, %	1.11	1.15	1.77
Histidine, %	1.10	1.19	2.51
Arginine, %	3.38	3.52	3.71
Proline, %	2.68	2.66	2.81
Anti-nutritional factors			
Glycinin, mg/g	128.95	2.58	-ND
β-conglycinin, mg/g	95.14	4.91	-ND
Trypsin inhibitor, mg/g	9.69	ND	-ND

*TCA-SP, Trichloroacetic acid soluble protein*.

**Table 2:** Ingredients and nutrient composition of three diets.

**Items**	**FM**	**ESBM-1**	**ESBM-2**
Ingredients			
Corn	71.49	70.52	68.88
Soybean meal	15.31	11.93	15.26
Soybean lecithin oil powder	4.00	4.00	4.00
Fish meal	4.00	2.00	–
ESBM (enzymatically treated soybean protein)	–	6.00	6.00
Dicalcium phosphate	0.01	0.32	0.57
Limestone	1.04	1.07	1.07
Salt	0.31	0.27	0.32
lysine	0.60	0.64	0.65
L-threonine	0.24	0.25	0.25
Premix^a^	3.00	3.00	3.00
Total	100.00	100.00	100.00
Nutrient composition			
Calculated			
Metabolizable energy MJ/kg	12.64	12.59	12.57
Digestible lysine %	1.00	1.00	1.10
Digestible methionine %	0.30	0.30	0.40
Digestible threonine %	0.53	0.53	0.53
Digestible tryptophan %	0.11	0.11	0.11
Crude protein %	16.95	17.03	16.98
Calcium %	0.68	0.68	0.68
Total phosphorus %	0.50	0.50	0.50
Available phosphorus %	0.28	0.28	0.28
Analyzed			
Dry matter %	87.36	87.73	87.68
Crude protein %	16.55	16.40	16.40
Ether extract %	5.02	4.74	4.46
Crude ash %	2.22	2.09	1.98
Neutral detergent fiber %	9.13	8.69	8.84
Acid detergent fiber %	3.26	3.13	3.17
Calcium %	0.66	0.64	0.65
Total phosphorus %	0.43	0.45	0.47

a *Provided the following per kg of diet: 12,000 IU VA as vitamin A acetate, 2,400 IU VD_3_ as vitamin D_3_, 40 mg VE as DL-α-tocopheryl acetate, 3 mg VK_3_ as menadione sodium bisulfate, 6 mg VB_1_, 9 mg VB_2_, 13 mg VB_6_, 19 mg D-pantothenic acid as calcium pantothenate, 40 mg nicotinic acid, 1 mg folic acid, 120 mg Fe as iron sulfate, 35 mg Mn as manganese sulfate, 75 mg Zn as zinc oxide, 12 mg Cu as copper sulfate, 1 mg I as potassium iodide, 0.3 mg Se as sodium selenite*.

### Sample Collection

On Days 14 and 28, ~10 mL blood samples from 1 pig (median BW) of each pen were collected *via* the jugular vein into vacuum tubes (Shenzhen Huachenyang Tech Co., Ltd., Guangdong, China). Serum was obtained by centrifuging blood at 3,000×g for 10 min at 4°C and was then stored at −20°C for further analysis. On Day 28, feces (~200 g) from each pen were collected and immediately frozen in liquid nitrogen and then stored at −80°C for microbiota analysis.

### Chemical Analysis of Experimental Diets and Measurement of Serum Parameters

The parameters of the 3 treatments were determined in terms of duplicate for dry matter (DM), crude protein (CP), ether extract, ash, calcium, and phosphorus, according to the procedures of AOAC (18). Neutral detergent fiber (NDF) and acid detergent fiber (ADF) were analyzed following the procedure of Van Soest et al. (19). Amino acids were assayed according to method described by Ma et al. (6). The detection method of trichloroacetic acid soluble protein (TCA-SP) was in according to Li et al.'s description (15). The concentrations of globulin, β-conglycinin and trypsin inhibitor were analyzed using ELISA kits (Longkefangzhou Bio-Engineering Technology Company, China), measured at 450 nm using a spectrophotometer.

The serum concentrations of 5-hydroxytryptamine (5-HT), diamine oxidase (DAO), D-lactate, interleukin-1β (IL-1β), IL-6, tumor necrosis factor α (TNF-α), nitric oxide (NO), immunoglobulin A (IgA), IgG, IgM, IgE, superoxide oxidase dismutase (SOD), malondialdehyde (MDA), cortisol, tri-iodothyronine (T3), thyroxine (T4), leptin, secretory curl associated protein 5 (sFRP-5), and caspase 3 (Casp-3) were determined using ELISA kits (Meimian Industrial Co., Ltd., Jiangsu, China), and measured at 450 nm using a spectrophotometer (800TSI, BioTek, USA) following the manufacturer's instructions.

### Fecal Microbiota Analysis

#### DNA Extraction

Total genomic DNA of each fecal sample was extracted using the OMEGA Soil DNA Kit (M5635-02) (Omega Bio-Tek, Norcross, GA, USA) following the manufacturer's instructions. Samples were stored at −20°C prior to further analysis. The quantity of extracted DNA was measured using a NanoDrop NC2000 spectrophotometer (Thermo Fisher Scientific, Waltham, MA, USA), OD260/280≥1.8 was defined as pure DNA. The quality of extracted DNA was measured with 1.2% agarose gel electrophoresis.

#### 16S rRNA Gene Amplicon Sequencing

The V3-V4 region of 16S rRNA genes was amplified using PCR with the forward primer 338F (5′-ACTCCTACGGGAGGCAGCA-3′) and the reverse primer 806R (5′-GGACTACHVGGGTWTCTAAT-3′). Sample-specific 7-bp barcodes were incorporated into the primers for multiplex sequencing. The PCR contained 5 μl of buffer (5×), 0.25 μl of Fast pfu DNA Polymerase (5 U/μl), 2 μl (2.5 mM) of dNTPs, 1 μl (10 uM) of each forward and reverse primer, 1 μl of DNA template, and 14.75 μl of ddH2O. Thermal cycling consisted of initial denaturation at 98°C for 5 min, 25 cycles of denaturation at 98°C for 30 s, annealing at 52°C for 30 s, and extension at 72°C for 45 s, with a final extension of 5 min at 72°C. The PCR amplicons were purified with Vazyme VAHTSTM DNA Clean Beads (Vazyme, Nanjing, China) and quantified using the Quant-iT PicoGreen dsDNA Assay Kit (Invitrogen, Carlsbad, CA, USA). The purified amplicons were pooled in equal amounts, and paired-end 2 × 250-bp sequencing was performed using the Illumina MiSeq platform (Illumina Inc., San Diego, CA, USA) with MiSeq Reagent Kit v3 at Shanghai Personal Biotechnology Co., Ltd. (Shanghai, China).

#### Sequence Analysis

QIIME2 (20) was used for sequence processing with slight modification according to the official tutorials (https://docs.qiime2.org/2019.4/tutorials/). Briefly, raw sequence data were demultiplexed using the demux plugin followed by primer cutting with the cutadapt plugin (21). Sequences were then quality filtered, denoised, and merged, and chimeras were removed using the DADA2 plugin (22). Non-singleton amplicon sequence variants (ASVs) were aligned with mafft (23) and used to construct a phylogeny with fasttree2 (24). Alpha diversity was evaluated by Chao1, Shannon, Simpson, Pielou's evenness, observed species, and Good's coverage. Beta diversity was evaluated based on the Bray-Curtis dissimilarity for principal coordinate analysis (PCoA), that were estimated using the diversity plugin with samples rarefied to 52,435 sequences per sample. Taxonomy was assigned to ASVs using the classify-sklearn naïve Bayes taxonomy classifier in the feature classifier plugin against the gg_13 database (25). Shared and unique ASVs among treatments were visualized by a Venn diagram using the R package “VennDiagram,” which was based on the occurrence of ASVs across treatments regardless of their relative abundance (26). All raw Illumina pair-end read data involved in the present study deposited in the NCBI Sequence read archive (SRA) database under accession number PRJNA 816316.

### Statistical Analysis

Data were analyzed using SPSS Statistics 21.0 software (SPSS Inc., Chicago, IL, USA). Shapiro-Wilk normality test and Levene's test were used to verify normality and homogeneity of variance, respectively. Data of growth performance and serum parameters were analyzed using ANOVA, statistical differences among treatments were determined by Tukey *post-hoc* test. Significant differences were defined as *P* ≤ 0.05, and a trend toward differences was defined as 0.05 < *P* ≤ 0.10. Sequence data analyses were mainly performed using QIIME2 and R packages (version 3.2.0). ASV-level alpha diversity indices include Chao1, Shannon index, Simpson index, Pielou's evenness observed species, and Good's coverage were calculated using the ASV table in QIIME2, Kruskal-Wallis rank sum test, and dunn's *post-hoc* test were used to verify statistical differences among treatments in α-diversity. Beta diversity was calculated based on the Bray-Curtis dissimilarity for principal coordinate analysis (PCoA), the significance of differentiation of microbiota structure among groups was assessed by PERMANOVA with 999 replicate permutations. Taxa abundances at the ASV levels were statistically compared among groups by MetagenomeSeq (occurrence not <0.3) and visualized as Manhattan plots. Differentially abundant taxa across treatments were detected using LEfSe analysis if the LDA score exceeded 2. Correlations between serum parameters and abundance of fecal microbiota were evaluated using Spearman correlation analysis.

## Results

### Growth Performance and Diarrhea Rate in Piglets That Consumed Different Sources of Dietary Protein

As shown in Table 3, ESBM2 increased BW of piglets at d14 and d 28 compared with FM and ESBM1 (*P* < 0.05). The ADG of piglets from the ESBM2 group significantly increased compared with the FM and ESBM1 groups from 1–14 d and 1–28 d (*P* < 0.05). The ADFI in the ESBM2 group was higher than those in the ESBM1 and FM groups from 1–14 d and 1–28 d (*P* < 0.05), and a significant difference was also observed between the ESBM1 and FM groups from 1–14 d (*P* < 0.05). The diarrhea rate of piglets in the ESBM2 group was decreased compared with the FM and ESBM1 groups during the whole experimental period (*P* < 0.05), and that of the ESBM1 group was decreased compared with the FM group from 1–14 d and 1–28 d (*P* < 0.05). No difference was observed in the F: G ratio among the 3 different dietary treatments.

**Table 3:** Effect of dietary protein source on growth performance and diarrhea in piglets.

**Items**	**FM**	**ESBM-1**	**ESBM-2**	**SEM**	***P-*value**
Initial BW, kg	7.17	7.19	7.20	0.11	0.95
Day 14 BW, kg	9.82^b^	10.14^b^	11.48^a^	0.22	<0.01
Day 28 BW, kg	17.07^b^	17.57^b^	19.29^a^	0.32	<0.01
1 to 14 d					
ADG, g	189.20^b^	210.76^b^	305.86^a^	17.30	<0.01
ADFI, g	254.57^c^	285.71^b^	403.75^a^	20.52	<0.01
F: G	1.35	1.36	1.32	0.03	0.08
Diarrhea rate, %	8.12^a^	5.53^b^	3.16^c^	0.27	<0.01
15 to 28 d					
ADG, g	517.73	530.45	557.54	38.75	0.18
ADFI, g	833.06	866.34	900.55	49.37	0.06
F: G	1.61	1.64	1.62	0.09	0.82
Diarrhea rate, %	4.02^a^	3.76^a^	1.94^b^	0.22	<0.01
0 to 28d					
ADG, g	353.47^b^	370.60^b^	431.70^a^	19.09	<0.01
ADFI, g	543.81^b^	576.02^b^	652.15^a^	28.04	<0.01
F: G	1.54	1.56	1.51	0.06	0.39
Diarrhea rate, %	6.07^a^	4.65^b^	2.55^c^	0.22	<0.01

*Results are presented as mean and SEM. Different letters (a, b, c) represent significant differences, P < 0.05. BW, body weight, ADG, average daily gain; ADFI, average daily feed intake*.

### Serum 5-HT, D-Lactate and DAO in Piglets That Consumed Different Sources of Dietary Protein

The serum concentrations of 5-HT in the ESBM1 and ESBM2 groups were significantly lower than those in the FM groups at d 14 and d 28, respectively (*P* < 0.05, Table 4). ESBM1 decreased the DAO content in serum compared with other two groups at d 14 and d 28 (*P* < 0.05). No difference was observed for D-lactate among three treatments.

**Table 4:** Effect of dietary protein source on serum parameters reflected mucosal permeability in piglets.

**Items**	**FM**	**ESBM-1**	**ESBM-2**	**SEM**	***P-*value**
14d					
5-HT, ng/mL	178.22^a^	143.95^b^	136.51^b^	8.38	<0.01
D-lactate, μmol/L	57.54	54.68	54.86	2.28	0.19
DAO, ng/mL	7.14^a^	5.20^b^	6.39^a^	0.47	<0.01
28d					
5-HT, ng/mL	179.81^a^	142.49^b^	135.32^b^	8.65	<0.01
D-lactate, μmol/L	57.00	53.82	54.41	2.47	0.20
DAO, ng/mL	6.93^a^	5.18^b^	6.41^a^	0.50	<0.01

*Results are presented as mean and SEM. Different letters (a, b, c) represent significant differences, P < 0.05. 5-HT, 5-hydroxytryptamine; DAO, diamine oxidase*.

### Serum Inflammatory Cytokines and Immunoglobulins in Piglets That Consumed Different Sources of Dietary Protein

According to the results in Table 5, the concentration of IL-6 in serum was decreased significantly in the ESBM1 group compared with the other 2 groups at d 14 and d 28 (*P* < 0.05), no difference was observed between the FM and ESBM2 groups at d 14 and d 28, respectively. The ESBM1 group had a lower IL-1β content at d 14 (*P* < 0.05), and the IgE level was higher in the ESBM1 group than in the FM and ESBM2 groups at d 14 and d 28 (*P* < 0.05). No differences were observed for TNF-α, NO, IgA, IgG and IgM among the 3 treatments at d 14 and d 28.

**Table 5:** Effect of dietary protein source on serum inflammatory cytokines and immunoglobulins in piglets.

**Items**	**FM**	**ESBM-1**	**ESBM-2**	**SEM**	***P-*value**
14d					
IL-1β, pg/mL	19.61^a^	18.79^b^	19.07^ab^	0.20	0.03
IL-6, pg/mL	100.90^a^	77.24^b^	99.47^a^	6.34	<0.01
TNF-α, pg/mL	219.33	227.93	228.38	12.08	0.52
NO, μmol/L	111.39	109.75	109.87	4.88	0.89
IgA, g/L	0.94	0.95	0.97	0.02	0.39
IgG, g/L	10.69	10.74	10.95	0.26	0.60
IgM, g/L	1.38	1.43	1.41	0.04	0.53
IgE, g/L	1.81^b^	2.06^a^	1.90^b^	0.06	<0.01
28d					
IL-1β, pg/mL	20.86	20.97	21.02	0.08	0.24
IL-6, pg/mL	100.22^a^	77.54^b^	99.27^a^	6.06	<0.01
TNF-α, pg/mL	223.98	226.53	229.40	10.97	0.79
NO, μmol/L	108.58	103.32	109.29	5.25	0.27
IgA, g/L	1.07	1.11	1.12	0.02	0.52
IgG, g/L	12.09	12.06	12.04	0.24	0.98
IgM, g/L	1.55	1.55	1.60	0.04	0.37
IgE, g/L	1.82^b^	2.06^a^	1.92^b^	0.08	<0.01

*Results are presented as mean and SEM. Different letters (a, b, c) represent significant differences, P < 0.05. IL-1β, interleukin 1β; IL-6, interleukin 6; TNF-α, tumor necrosis factor α; NO, nitric oxide; IgA, immunoglobulin A; IgG, immunoglobulin G; IgM, immunoglobulin M; IgE, immunoglobulin E*.

### Oxidative Status-Related Serum Parameters in Piglets That Consumed Different Sources of Dietary Protein

As shown in Table 6, ESBM2 increased serum SOD compared with the other two groups at d 14 and d 28, respectively (*P* < 0.05), the concentration of SOD in ESBM1 group was higher than that in FM at d 14 and d 28 (*P* < 0.05). ESBM2 treatment resulted in a lower MDA content than in the other 2 groups at d 14 and d 28 (*P* < 0.05).

**Table 6:** Effect of dietary protein source on oxidative status related serum parameters in piglets.

**Items**	**FM**	**ESBM-1**	**ESBM-2**	**SEM**	***P-*value**
14d					
SOD, ng/mL	40.19^c^	50.94^b^	64.08^a^	4.52	<0.01
MDA, nmol/mL	24.00^a^	24.83^a^	20.87^b^	0.45	<0.01
28d					
SOD, ng/mL	38.65^c^	50.94^b^	63.78^a^	5.58	<0.01
MDA, nmol/mL	24.56^a^	24.11^a^	20.60^b^	0.56	<0.01

*Results are presented as mean and SEM. Different letters (a, b, c) represent significant differences, P < 0.05. SOD, super oxidase dismutase; MDA, malondialdehyde*.

### Serum Hormone Levels in Piglets That Consumed Different Sources of Dietary Protein

As shown in Table 7, the serum concentration of cortisol in the ESBM1 group decreased compared with those in the FM and ESBM2 groups at d 14 and d 28 (*P* < 0.05), FM had lower level of cortisol than that in ESBM2 at d 28 (*P* < 0.05). ESBM1 and ESBM2 decreased T3 compared with FM at d 14 and d 28 (*P* < 0.05). T4/T3 ratio was lower in FM group than that in ESBM1 and ESBM2 (*P* < 0.05). The concentration of leptin in the serum was lower in the ESBM2 treatment than in the other two treatments at d 28 (*P* < 0.05), and ESBM2 decreased serum leptin level compared with FM (*P* < 0.05) and tended to decrease compared with ESBM1 (*P* = 0.099) at d 14, respectively. No difference was found for T4.

**Table 7:** Effect of dietary protein source on serum hormone level in piglets.

**Items**	**FM**	**ESBM-1**	**ESBM-2**	**SEM**	***P-*value**
14d					
Cortisol, ng/mL	140.12^a^	113.71^b^	146.94^a^	4.17	<0.01
T3, pmol/L	10.41^a^	7.57^b^	7.80^b^	0.54	<0.01
T4, nmol/L	178.33	179.27	180.05	42.42	0.99
T4/T3	17.12^b^	23.68^a^	23.07^a^	0.70	<0.01
Leptin, pmol/L	13.72^a^	13.10^ab^	12.25^b^	0.52	<0.01
28d					
Cortisol, ng/mL	140.60^b^	115.37^c^	154.38^a^	4.83	<0.01
T3, pmol/L	10.73^a^	7.50^b^	7.98^b^	0.49	<0.01
T4, nmol/L	178.95	179.58	176.60	44.05	0.99
T4/T3	16.68^c^	23.95^a^	22.14^b^	0.59	<0.01
Leptin, pmol/L	13.74^a^	13.44^a^	11.94^b^	0.34	<0.01

*Results are presented as mean and SEM. Different letters (a, b, c) represent significant differences, P < 0.05. T3, tri-iodothyronine; T4, thyroxine*.

### Serum SFRP-5 and Casp-3 in Piglets That Consumed Different Sources of Dietary Protein

As shown in Table 8, the serum concentration of sFRP-5 in the ESBM1 group was increased compared with those in the FM and ESBM2 groups at d 14 and d 28 (*P* < 0.05). The ESBM1 group decreased Casp-3 compared with FM and ESBM2 at d 14 and d 28 (*P* < 0.05). ESBM2 had higher serum Casp-3 than that in FM at d 14 and d 28 (*P* < 0.05).

**Table 8:** Effect of dietary protein source on Leptin, sFRP-5 and Casp-3 in serum of piglets.

**Items**	**FM**	**ESBM-1**	**ESBM-2**	**SEM**	***P-*value**
14d					
sFRP-5, ng/mL	17.11^b^	20.01^a^	16.07^b^	0.78	0.02
Casp-3, pmol/mL	34.32^b^	30.45^c^	36.74^a^	0.55	<0.01
28d					
sFRP-5, ng/mL	17.31^b^	18.75^a^	16.43^b^	0.72	<0.01
Casp-3, pmol/mL	34.84^b^	30.11^c^	36.80^a^	0.57	<0.01

*Results are presented as mean and SEM. Different letters (a, b, c) represent significant differences, P < 0.05. sFRP-5, secretory curl associated protein 5; Casp-3, caspase 3*.

### Microbiota Composition in the Feces of Piglets fed Different Sources of Protein

**Figure 1:**
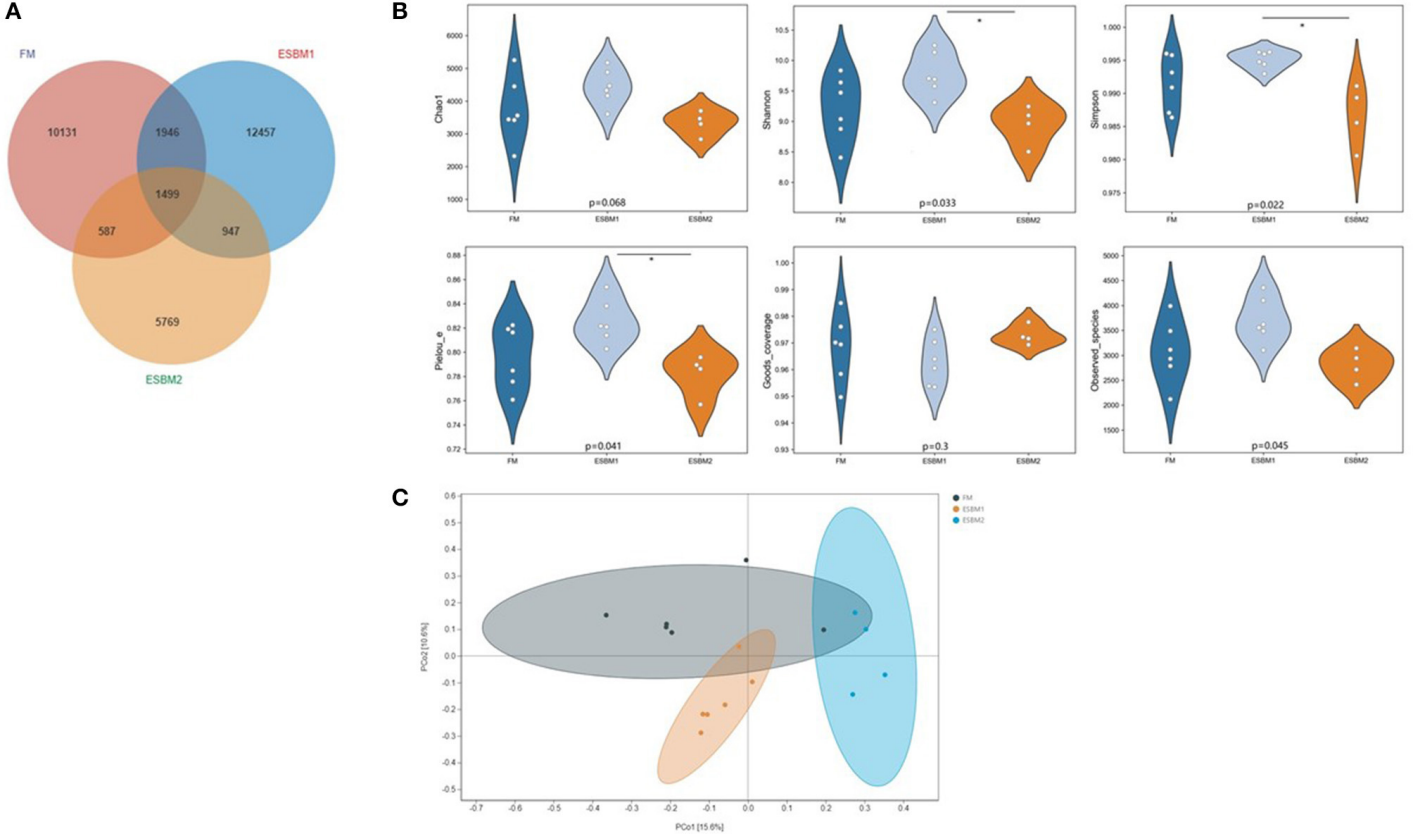
Microbiota richness and diversity in feces. **(A)** Venn diagram based on ASV level. **(B)** alpha diversity indices of 3 treatments, showed as box plot of Chao1, Shannon, Simpson index, Pielou's evenness, Good's coverage, and Observed species. The results were analyzed by Kruskal-Wallis H test. **(C)** principal coordinate analysis (PCoA) based on Bray_Curtis distance. Significant difference was observed among three groups, difference between FM and ESBM1 (*P* = 0.005), FM and ESBM2 (*P* = 0.009), ESBM1 and ESBM2 (*P* = 0.004) (*n* = 6 for FM and ESBM1 groups and *n* = 4 for ESBM2 group).

According to the Venn diagram analysis of ASVs, 1499 shared ASVs were identified, and 10131, 12457, 5769 unique ASVs identified in the FM, ESBM1 and ESBM2 groups, respectively (Figure 1A). α-diversity was evaluated to demonstrate the richness and evenness of species. The indices of Shannon, Simpson and Pielou's_e increased in ESBM1 group compared with ESBM2 group (*P* < 0.05) (Figure 1B). The differences of microbiota composition were presented as β-diversity index of PCoA (Bray-Curtis distance). Significant difference of β-diversity was observed among three groups (Adonis; difference between FM and ESBM1, *P* = 0.005; FM and ESBM2, *P* = 0.009; ESBM1 and ESBM2, *P* = 0.004) (Figure 1C).

**Figure 2:**
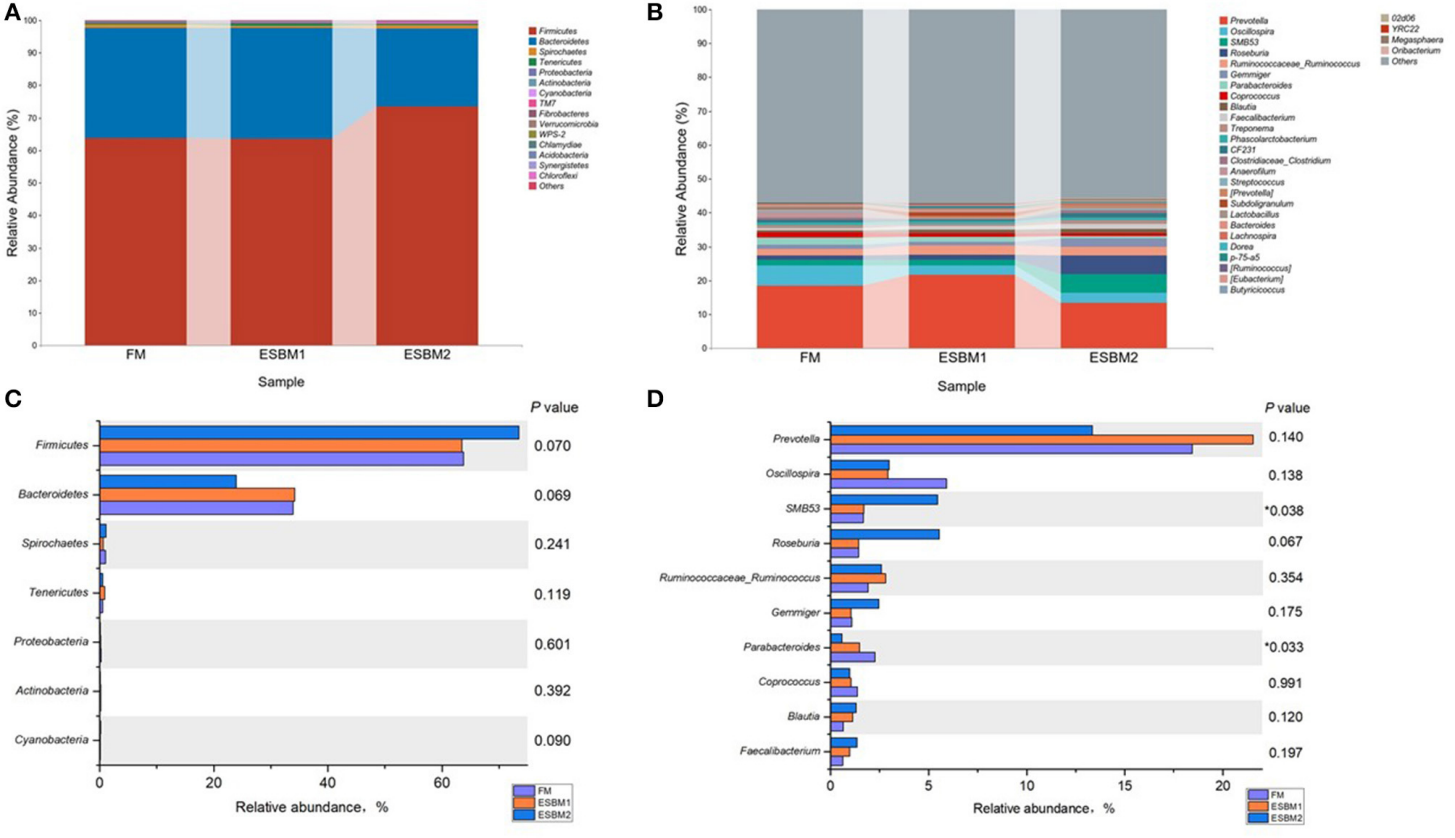
Microbiota composition in feces of piglets fed different source of protein. Abundance of microbiota at **(A)** phylum level, **(B)** genus level. Differences of relative abundance were tested using Kruskal-Wallis method at phylum **(C)** and genus **(D)** level, ^*^ marked *P* < 0.05 (*n* = 6 for FM and ESBM1 groups and *n* = 4 for ESBM2 group).

In the feces of piglets, Firmicutes and Bacteroidetes were the two predominant phyla, accounting for more than 63 and 23% of the total microbiota abundance, respectively (Figure 2A). No differences were found in the relative abundance of top seven phyla among three treatments. There were tendency toward increasing the abundance of Firmicutes (*P* = 0.070) and decreasing Bacteroidetes (*P* = 0.069) in ESBM2 group compared with FM and ESBM1, respectively (Figure 2C). The top 30 most abundant genera are shown in Figure 2B. *Prevotella* was the most favorable genus in the feces of piglets fed different diets, comprising 18.43, 21.53, and 13.34% of the total genera in the FM, ESBM1 and ESBM2 groups, respectively. Genus *SMB53* increased in ESBM2 group compared with FM and ESBM1, and *Parabacteroides* decreased in ESBM2 compared with FM (*P* < 0.05). ESBM2 tend to increase the abundance of *Roseburia* compared with the other two groups (*P* = 0.067, Figure 2D).

**Figure 3:**
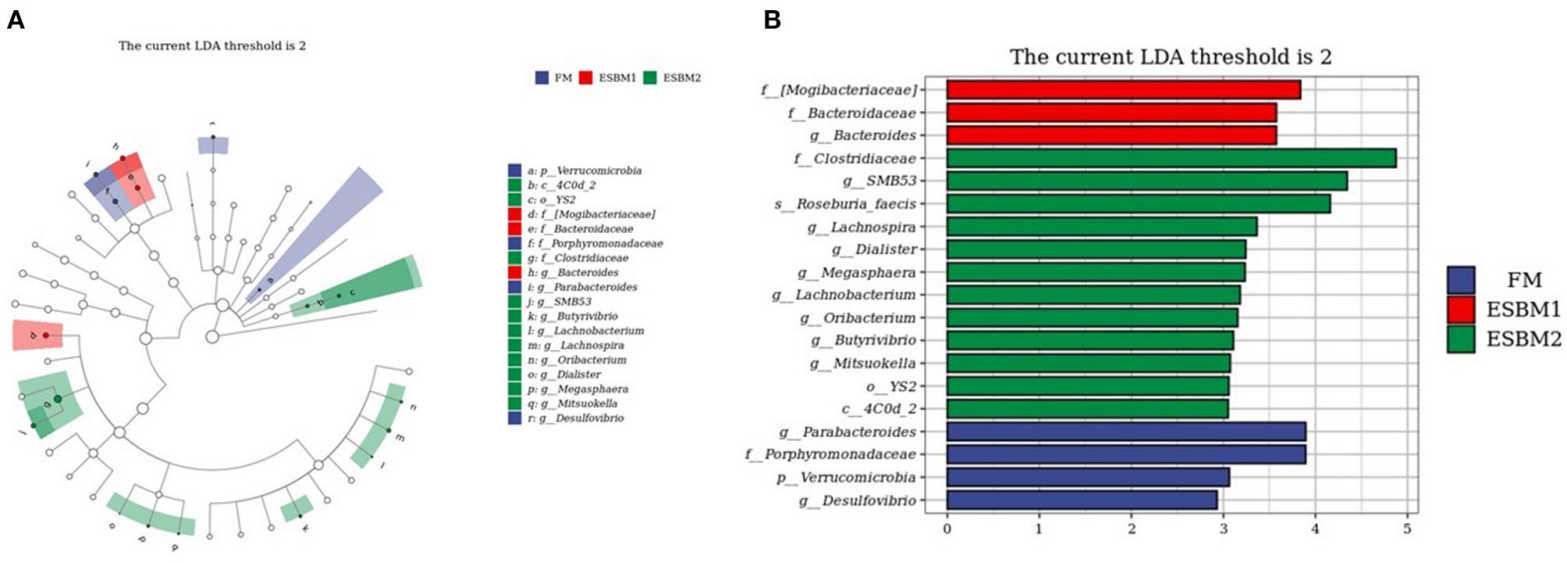
Linear discriminant analysis effect size (LEfSe) analysis among treatments. Kruskal-Wallis rank-sum test were used for comparison, genera with significant differences (*P* < 0.05) were shown in histograms **(A)** and cladogram **(B)**. The linear discriminant analysis (LDA) threshold was 2 (*n* = 6 for FM and ESBM1 groups and *n* = 4 for ESBM2 group).

LEfSe analyses was used to identify the specific bacteria among three treatments. The genera *SMB*53, *Lachnospira, Dialister, Megasphaera, Lachnobacterium, Oribacterium, Butyrivibrio, Mitsuokella* were enriched in the ESBM2 group, *Bacteroides* enriched in the ESBM1 group, *Parabacteroides* and *Desulfovibrio* enriched in the FM group (Figure 3).

**Figure 4:**
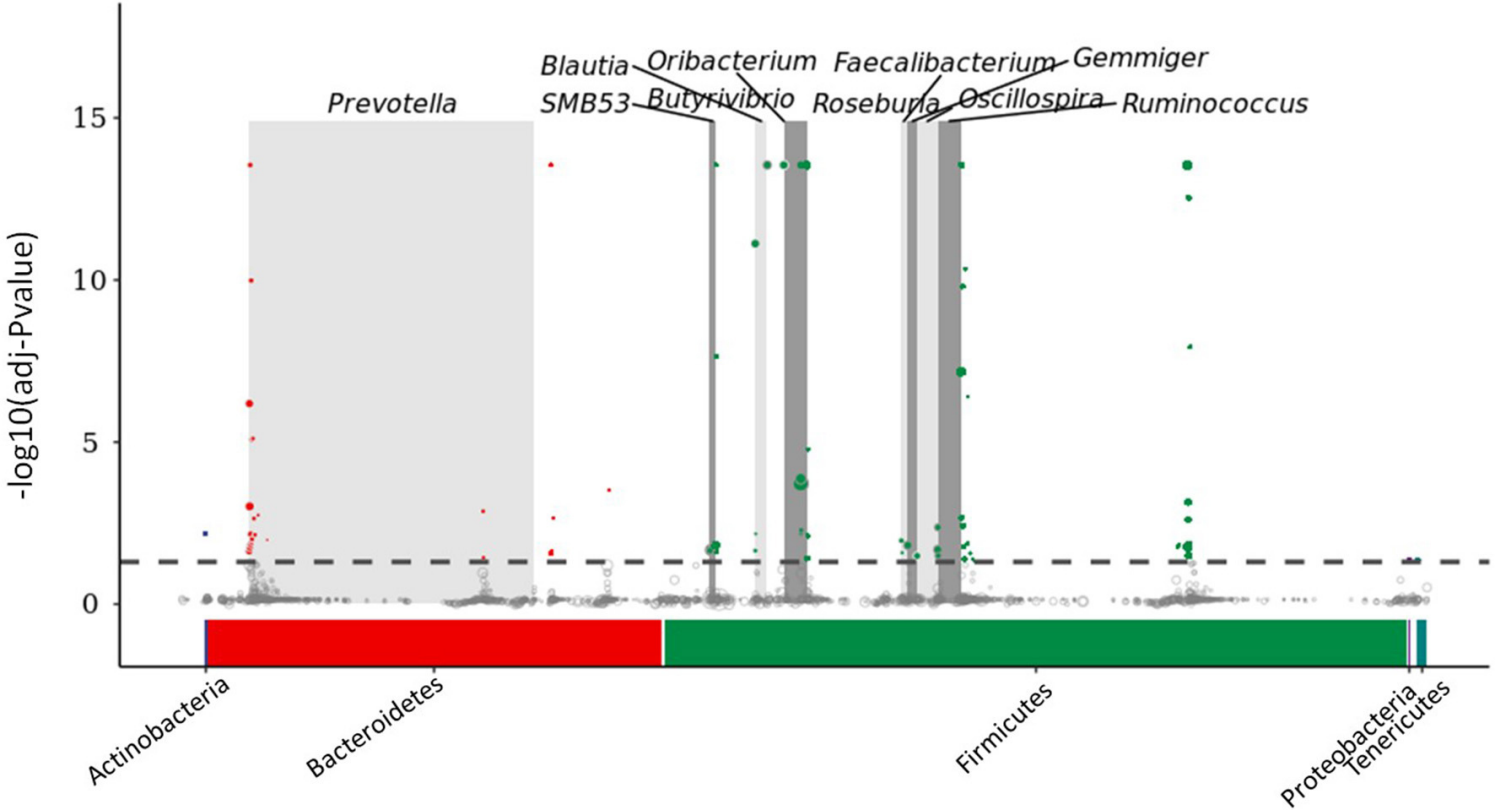
MetagenomeSeq analysis of fecal enriched core microbe between two treatments. ESBM2 to FM. Each circle represented an ASV, and the size of the circle was calculated as log2 (CPM/n), (CPM, copy per million; n, sample number). Significantly enriched ASVs in ESBM 2 compared with FM were marked as colored circles above dashed line. Gray boxes were used to denote the different taxonomic groups (*n* = 6 for FM groups and *n* = 4 for ESBM2 group).

MetagenomeSeq analyses were performed by comparing abundance at the ASV level between ESBM2 and FM treatments. Firmicutes and Bacteroidetes enriched in the ESBM2 group at phylum level, *Prevotella, SMB53, Ruminococcus, Gemmiger, Oscillospira, Faecalibacterium, Oribacterium, Blautia, Butyrivibrio*, and *Roseburia* enriched in the ESBM2 group at genus level (Figure 4).

### Correlations Between the Intestinal Microbiota and Serum Parameters

**Figure 5:**
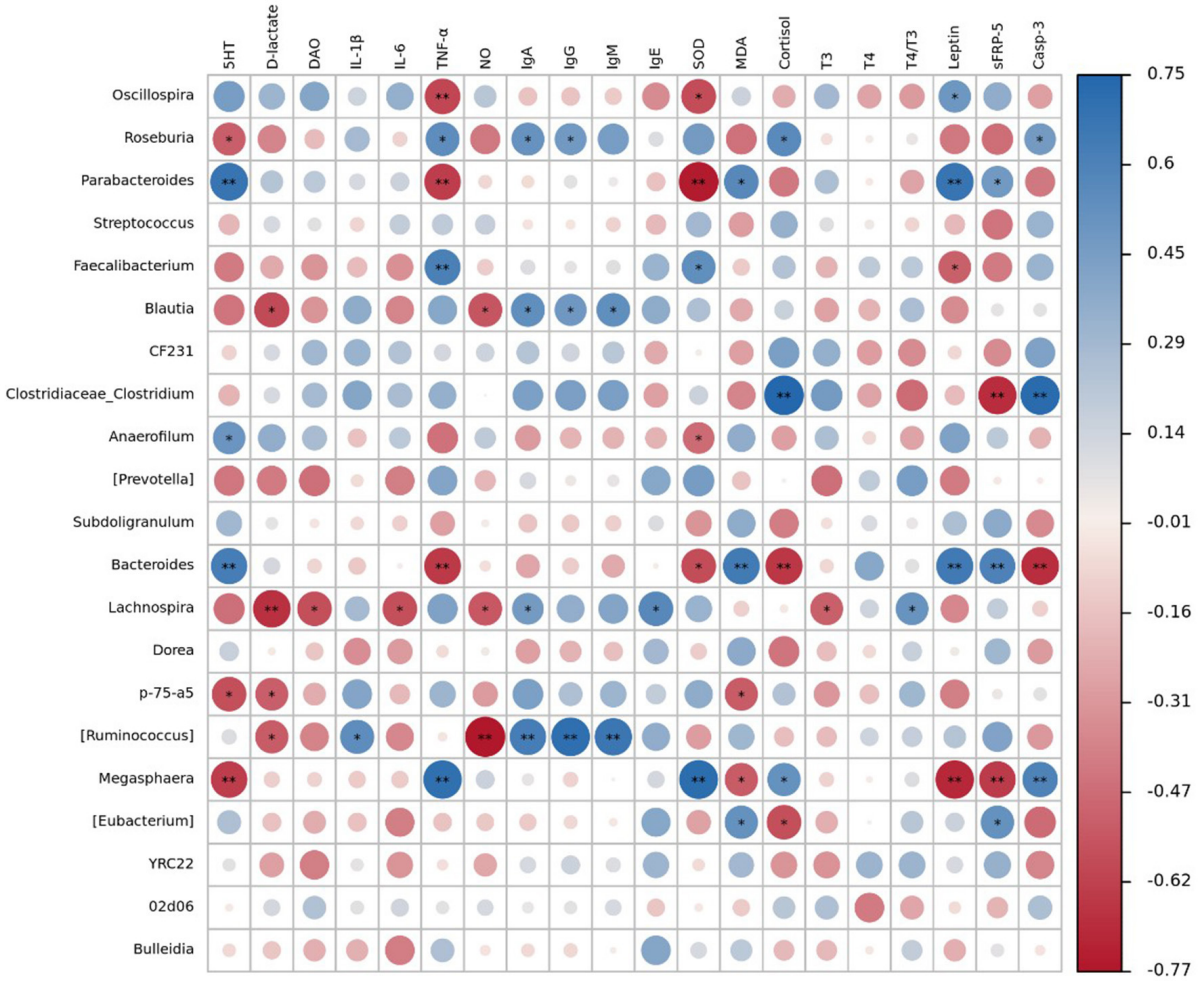
The Spearman correlation between serum biochemical parameters and fecal microbiota. Significances are marked as ^*^ and ^**^ which in accordance with 0.01 ≤ *P* < 0.05 and *P* < 0.01, respectively. Blue circle represents positive correlation and red circle represents negative correlation (*n* = 6 for FM and ESBM1 groups and *n* = 4 for ESBM2 group).

Spearman correlation analysis was employed to investigate the correlations between serum immune-related, mucosal permeability-related, and oxidative stress-related biochemical indicators and fecal microbiota. The abundance of *Oscillospira* was negatively associated with TNF-α and SOD, and positively associated with leptin (*P* < 0.05). The abundance of *Roseburia* was negatively correlated with 5-HT, and positively correlated with TNF-α, IgA, IgG, cortisol, and Casp-3 (*P* < 0.05). The abundance of *Parabacteroides* was positively associated with 5-HT, leptin, MDA and sFRP-5, and negatively correlated with TNF-α and SOD (*P* < 0.05). The abundance of *Faecalibacterium* was positively correlated with TNF-α and SOD, and negatively associated with leptin (*P* < 0.05). The abundance of *Blautia* and *Ruminococcus* was positively correlated with IgA, IgG, and IgM, but negatively correlated with NO and D-lactate (*P* < 0.05). The abundance of *Clostridiaceae_Clostridium* was positively correlated with Cortisol and Casp-3, and negatively correlated with sFRP-5 (*P* < 0.05). The abundance of *Anaerofilum* was positively and negatively correlated with 5-HT and SOD, respectively (*P* < 0.05). The abundance of *Bacteroides* was positively correlated with 5-HT, MDA, leptin, and sFRP-5, and negatively correlated with TNF-α, cortisol, Casp-3, and SOD (*P* < 0.05). The abundance of *Lachnospira* was negatively correlated with D-lactate, DAO, IL-6, NO, T3, and positively associated with IgA, IgE, and T4/T3 (*P* < 0.05). As shown in the heatmap, the abundance of *p*-75-*a*5 was found to be negatively correlated with 5-HT, D-lactate, and MDA (*P* < 0.05). The abundance of *Megasphaera* negatively correlated with 5-HT, leptin, sFRP-5, and MDA, but positively correlated with TNF-α, SOD, cortisol, and Casp-3 (*P* < 0.05). The heatmap also revealed a negative correlation between the abundance of *Eubacterium* and Cortisol, but a positive correlation between the abundance of *Eubacterium* and MDA, sFRP-5 (*P* < 0.05) (Figure 5).

## Discussion

Bioprocessing plant protein has been considered an economical and well-performing fish meal alternative. Along with large-scale industrial enzyme development, highly efficient, more stable and various kinds of enzymes are available for bioprocessing treatment, meaning that more target chemical bonds can be cleaved, and the inner antinutritional factors, such as antigen proteins, can be structurally modified or cut into small pieces; therefore, antigen proteins pose less threat to the gastrointestinal tracts of animals. In present study, ESBM treated with compound enzyme has reduced over 94% antigen proteins. Regarding the growth performance data shown in the present study, we found that piglets fed only ESBM had higher ADG and ADFI than those fed only FM or FM+ESBM. The findings are partially in line with the results of Ma et al. (6), which demonstrated that piglets fed diet with 9% ESBM in both phases (0–14 d and 14–28 d) had higher ADG than pigs fed FM (contained 7.38% in diet) or ESBM in one-phase (0–14 d). The possible reason may cause by different levels of ESBM and FM used in our experiment. Also, the ESBM used in this study was processed in different ways with various enzymes, the TCA-SP was 18.98%, which means more small peptides are available in soluble part of soybean protein. Multiple small peptides were crucial in the product, which has great potential to improve the efficiency of growth in weaned piglets (27). Yun et al. (28) reported that replacing 6% SBM with equal amount of fermented soybean protein (FSP) or FM improved the ADG of early-weaned pigs compared with pigs fed with 15% SBM, during d 0–7, the FM group showed better performance compared with FSP, but after d 14, the growth performance was similar between the two groups. In this research, pigs fed ESBM diets had a lower diarrhea rate than pigs fed only FM, which is in accordance with the results of Ma et al. (6). In Ma's report, two-phase ESBM consumption had the lowest diarrhea rate in both phases compared with one-phase ESBM and FM consumption (6). The lower diarrhea in the ESBM2 group in present study may attribute to the decreased 5-HT content in serum compared with FM. Over 90% of 5-HT is synthesized, stored and released from enterochromaffin cells in the intestinal mucosa, which function as sensory transducers responding to chemical and mechanical stimuli in the lumen. Mucosal 5-HT can promote intestinal inflammation, causing diarrhea. A large concentration of 5-HT in serum reflects increasing intestinal permeability and excessive intrinsic peristalsis, segmentation and secretion (29).

Intestinal mucosal barrier function plays a crucial role in maintaining intestinal morphology and homeostasis by preventing the invasion of pathogens and other harmful substances (27, 30, 31). Intestinal permeability has been correlated with some serum parameters, such as D-lactate and DAO (9). Weaning is a stressful event that directly damages intestinal integrity, increased the serum concentrations of D-lactate, DAO and 5-HT compared with suckling treatment, moreover, soy protein antigens glycinin and β-conglycinin can aggravate this weaning stress through increasing even more D-lactate, DAO and 5-HT than that in weaned piglets without soy protein antigens consumption (32). In this study, 5-HT decreased in piglets fed ESBM1 and ESBM2 diets, both diets contained 6% ESBM. The concentration of serum DAO was lower in ESBM1 group, and similar between ESBM2 and FM groups, indicating the advantage of ESBM consumption in maintaining or even enhancing intestinal barrier function. In our research, the antigen protein of glycinin and β-conglycinin in ESBM decreased 98 and 94.84% compared with SBM, respectively. It may explain less intestinal barrier damage caused by antigen protein. The same results were also supported by Zhang and Piao (9), who reported that pigs fed diets with 15.1 and 7.5% ESBM in two phase decreased serum DAO at d 28 compared with FSBM and HWP.

β-conglycinin has been demonstrated to be a food-stimulated IgE-mediated factor that increases IgE secretion and cause allergy (33). Wang et al. (33) found that the serum IgE increased significantly after direct injection with a high dose of β-conglycinin. In the present study, the serum concentration of IgE showed no difference between the FM and ESBM-only groups but was significantly higher in the FM+ESBM group. The reason for the unchanged serum IgE content between FM and ESBM2 groups may attribute to the reduction of β-conglycinin content in ESBM in this experiment. However, the causation of different results showed in ESMB1 and ESBM2 was needed to further explore. NO in serum and some specific organs plays a key role in indicating inflammatory and oxidative status (34). When facing pathogen attack, macrophages and neutrophils of the innate immune system produce a large amount of NO to eliminate invasion through reaction with peroxide anions to produce peroxynitrite, which aggravates oxidative damage (35, 36). Piglets treated with β-conglycinin and glycinin had significantly increased serum NO contents (32). Oxidative stress also increases serum NO levels (34, 37). The present study demonstrated that dietary treatments with FM or ESBM did not affect NO content. It was indicated that the ESBM used in our study generated fewer inflammatory reactions caused by soya antigens existed in unprocessed SBM, according to the results demonstrated by Cao (32).

The equilibrium between oxidation and antioxidation in the body plays an important role in maintaining animal health, which may deteriorate by losing innate antioxidative molecules to defend the body from attack by overproducing ROs (34). SOD is one of the main endogenous antioxidant enzymes that inhibits and blocks free radical reactions and reduces the production of the free radical metabolite MDA (38–40). In this study, ESBM increased the serum concentrations of SOD in both the ESBM+FM and ESBM-only groups compared with FM treatment. Similar results were also found in Ma et al.'s (6) report that FM and ESBM increased the contents of SOD compared with the SBM group on both d 14 and d 28 and that ESBM also showed higher SOD content than FM on d 28. A reduction of MDA was also observed in the ESBM-only group compared with the other two groups. MDA is a secondary product of lipid peroxidation that damages cells and eventually leads to cell death (41). The serum concentration directly reflects the degree of lipid oxidative damage (42). Previous study demonstrated that His-containing peptides hydrolyzed from soybean proteins acted as metal-ion chelators, active-oxygen quenchers and hydroxyl-radical scavengers. The diet contained 4.5 g black soy peptides daily could increase plasma SOD and reduce MDA in human (43). This indicated that the changes of SOD and MDA may attribute to the small peptide content in ESBM.

Serum inflammatory cytokines are essential indicators of the inflammatory response (44). Proinflammatory cytokines are crucial factors in regulating the inflammatory response, and the levels of these cytokines, including IL-1β, IL-6, and TNF-α, reflect the inflammatory status of animals (9, 45, 46). In our study, we found FM+ESBM treatment significantly decreased the content of IL-6 compared with FM or ESBM consumption alone. Meanwhile, we also observed that FM+ESBM treatment increased sFRP-5 content compared with each dietary protein treatment alone. It has been suggested that sFRP5 is an anti-inflammatory factor inversely correlated with proinflammatory cytokines such as IL-1β, IL-6, and TNF-α in human studies. Secreted Frizzled-Related Proteins (SFRPs) are supposed to antagonize Wnt signaling, and Wnt5a can serve as an activator to macrophages, which produce proinflammatory cytokines according to a previous study (47–49). However, the phenomenon that ESBM plus FM reduced inflammation in this study needed to be confirm by further research.

Weaning stress could induce the activation of the hypothalamic–pituitary–adrenal (HPA) axis and the secretion of glucocorticoids (e.g., cortisol). Dietary ingredients that modulate neurobehavior are strongly related to altered neurotransmitter signaling systems, including the GABAergic, serotonergic, and dopaminergic systems (50). Decreasing salivary cortisol was found to be associated with higher growth performance after supplementation with tryptophan in the diet and may be the result of reduced stress (51). A similar result was also found in a study by Koopmans et al. (52), who observed a decrease in plasma cortisol by feeding surplus dietary tryptophan to pigs, and reduced stress and the long-term hormonal response to stress. But different finding was included in other report, infants with food protein-induced enterocolitis syndrome increased serum cortisol by oral challenging with cow's milk protein compared with that measured before oral food challenge (53). In the present study, FM+ESBM reduced cortisol content compared with the other two dietary treatments at d 14 and d 28, and FM decreased cortisol level compared with ESBM-only groups at d 28. A very limited information on the relation between dietary protein and serum cortisol is available. Dietary protein could be a potential impact factor to serum cortisol, and this need to be elucidated in future.

Evidence has shown that leptin negatively relates to appetite by increasing the frequency of action potentials in anorexigenic proopiomelanocortin (POMC) neurons, reducing inhibition by local orexigenic neuropeptide-Y/GABA neurons and depolarizing through a non-specific cation channel (54). In a previous study, dietary protein restriction increased leptin content compared with a basal diet (47). Our results showed that only ESBM consumption decreased serum leptin content, which may be reasons in consistent with the high growth performance in the ESBM2 group.

Tri-iodothyronine (T3) and thyroxine (T4) are essential for animal growth. Dietary protein restriction had no effect on the contents of T3 and T4 (47). Wang et al. (55) reported that dietary protein sources within FM, FSBM or a complex had no influence on the T3 and T4 levels of broilers at 42 d. In this study, FM significantly increased the T3 content. Data have rarely been evaluated to measure the fluctuation of serum triiodothyronine and thyroxine, which reflect the metabolism and stress status of pigs fed different dietary proteins. Further research is needed to assess the effects of protein source on serum hormones.

Caspase-3 catalyzes protein degradation during the apoptotic process, and decreasing Casp-3 gene expression protects animals from stress-related apoptosis (55). Plant proteins, such as soya protein-containing antigens, enhance the serum Casp-3 content of piglets (32). Chen et al. (3) also observed increased expression of the Casp-3 gene in cells exposed to β-conglycinin both *in vivo* and *in vitro*. In our study, FM+ESBM treatment had the lowest level of serum Casp-3, FM treatment was the second, and ESBM-only was relatively higher, considering that there may be an association between apoptosis and inflammation, as explained by similar comparative trends in IL-6 and Casp-3, but need further investigation.

Based on our current understanding, the microbial community is closely related to host feeding habits and physiological status, and will be inevitably changed by ingredients ingested from different sources. The gut microbiota is very important in maintaining healthy intestinal physiological structure, mucosal immunity and digestibility, and adapt to ingested substrates. Bacteria inhabit in the gut and express an array of enzymes that can degrade complex dietary substrates whose components cannot be digested by endogenous animal enzymes (56). Dietary components also play a vital role in modulate gut microbiota, and serve as a substrate for bacterial metabolism (57). In present study, we demonstrated the different microbiota composition reflected by β-diversity, which consolidated the effectiveness of dietary protein sources on the gut microbiota composition. Piglets fed FM+ESBM showed higher α-diversity according to the Chao1, Shannon, Simpson, and Pielou's evenness indices, which indicated that combined animal and plant protein tended to increase the richness and evenness of gut microflora.

Firmicutes and Bacteroidetes were the dominant phyla accounting for more than 63 and 23% of the total bacteria abundance in three dietary treatments in our results, respectively. The bacteria composition in this study was in accordance with the typical porcine microbiota colonization patterns described by Holman et al. (58), who demonstrated that Firmicutes and Bacteroidetes together account for 64–97% of total sequences across all ages. The relative abundance of Firmicutes in post-weaning piglets (14 days after weaning) increased from 44 to 67% compared with pre-weaning period (2 days after birth) (57). In this research, the relative abundance of Firmicutes tended to increase in piglets fed only ESBM, this may attribute to ESBM that is a plant-based protein source, accelerated the change of gut microbiota toward maturation, and adapt earlier to digest the plant-based feed. However, when ESBM was compared with other plant-based source hydrolyzed wheat protein (HWP) and fermented soybean meal (FSBM), the abundance of Bacteroidetes increased in ESBM treatment accompany by increased growth performance (9). This indicated that fecal microbiota was not only affected by dietary animal-based or plant-based protein sources, but also by which plant it applied and how they were processed.

Pigs early fed plant-based feed before weaning accelerated the formation and stabilization of gut microbiota, which may mark as microbial maturation of young animals. *Prevotella, Roseburia, Faecalibacterium, Ruminococcus, Megasphaera, Catenibacterium*, and *Subdoligranulum* were considered as representative microbiota for a stable and mature post-weaning microbiota (57). In present study, we found *Prevotella, Roseburia, Faecalibacterium, Ruminococcus*, and *Megasphaera* enriched more in feces of piglets fed only plant-based ESBM than FM, which means ESBM without fishmeal could accelerate the maturation of gut microbiota. Choudhury et al. (57) demonstrated that *Bacteroides* considered as pre-weaning prevalent genera, but lost fast in early fed piglets. In our study, *Bacteroides* was found enrich in ESBM1 group (contained both ESBM and FM in diet) compared with pigs fed only FM and ESBM, the possible reason may be that fish meal content in diet is well metabolic substrate for *Bacteroides*, but further study needed to be conducted.

The relative abundance of *Parabacteroides* decreased in feces of piglets fed only ESBM compared with FM. According to Spearman correlation analysis, the abundance of *Parabacteroides* was negatively correlated with SOD. In previous study, the abundance of *Parabacteroides* was found decreased when gilts were supplied with organic selenium, and the abundance of *Parabacteroides* inversely associated with antioxidant capacity (59). *Megasphaera* and *Faecalibacterium* enriched in ESBM2 also showed strong positive correlation with SOD in our study. This observation may support that ESBM increased antioxidant capacity through modulate typical bacterial species. We observed *Lachnospira* enriched in ESBM-only group, the abundance of *Lachnospira* were negatively correlated with serum concentration of D-lactate, DAO, IL-6, and NO. Inconsistently, a previous research was conducted to evaluate the high-fructose intake in rats on gut microbiota and inflammatory response, it was considered that fructose overconsumption induced inflammation and metabolic disorders owing to changes in gut microbiota and enhancing intestinal permeability, the results demonstrated a positive connection between inflammatory response, intestinal permeability and *Lachnospira* in rats with high fructose intake (60). The possible reason may be animals are in a highly inflammatory status with large amount of fructose intake. The piglets in this study were in normal and healthier status. Rats are different from pigs as well. So, it would be interesting to investigate the correlation between different bacterial species and inflammatory factors under different inflammatory status.

## Conclusions

Result from our study indicate that replacing fish meal half or all by enzymatically treated soybean protein in diets shows equal or even better growth performance, accompanied by decreasing diarrhea rate. This may be a result of the positive effect of ESBM on serum concentrations of indicators for intestinal integrity, antioxidant capacity. The different intestinal microbiota composition with more enriched microbial species that fit for plant-based diet digestion, may be reasons for better performance of piglets only fed ESBM, either. However, ESBM only or ESBM plus FM showed some differences on some serum indicator for immunity, stress, and inflammatory cytokines, further research is needs to determine the mechanism of ESBM in exerting positive effect on pig performance.

## Data Availability Statement

The data presented in the study are deposited in the NCBI BioProject repository, accession number PRJNA816316, https://www.ncbi.nlm.nih.gov/sra/?term=PRJNA816316.

## Ethics Statement

The animal study protocol was approved by the Institutional Ethics Committee of Shenyang Agricultural University Institutional Animal Care and Use Committee (pro-tocol code 2021042567).

## Author Contributions

NY and ZQ: conceptualization. NY: methodology, formal analysis, investigation, and writing—original draft preparation. NY and HL: software. NY, ZQ, and XY: validation. ZQ: resources. XY: data curation, visualization, supervision, and project administration. ML, YH, JL, and XL: writing—review and editing. XY and ZQ: funding acquisition. All authors have read and agreed to the published version of the manuscript. All authors contributed to the article and approved the submitted version.

## Funding

This research was funded by Liaoning Revitalization Talents Project, Grant Numbers: XLYC1902083 and XLYC1902024 and Liaoning Distinguished Professor Project, Grant Number: 01086217001.

## Conflict of Interest

ZQ was employed by Liaoning Complete Biotechnology Co., Ltd. The remaining authors declare that the research was conducted in the absence of any commercial or financial relationships that could be construed as a potential conflict of interest.

## Publisher's Note

All claims expressed in this article are solely those of the authors and do not necessarily represent those of their affiliated organizations, or those of the publisher, the editors and the reviewers. Any product that may be evaluated in this article, or claim that may be made by its manufacturer, is not guaranteed or endorsed by the publisher.
